# ERCC1 Cys8092Ala and XRCC1 Arg399Gln Polymorphisms Predict Progression-Free Survival after Curative Radiotherapy for Nasopharyngeal Carcinoma

**DOI:** 10.1371/journal.pone.0101256

**Published:** 2014-07-15

**Authors:** Hekun Jin, Xiaoxue Xie, Hui Wang, Jun Hu, Feng Liu, Zhigang Liu, Jumei Zhou, Yingying Zhang, Xuping Xi, Bingqiang Hu, Yuping Liao, Jingtian Tang

**Affiliations:** 1 Department of Radiation Oncology, Xiangya Hospital, Central South University, Changsha, China; 2 Department of Radiation Oncology, Hunan Provincial Tumor Hospital & Affiliated Tumor Hospital of Xiangya Medical School, Central South University, Changsha, China; 3 Department of Pathology, Hunan Provincial Tumor Hospital & Affiliated Tumor Hospital of Xiangya Medical School, Central South University, Changsha, China; 4 Key Laboratory of Particle and Radiation Imaging, Ministry of Education, Institute of Medical Physics and Engineering, Department of Engineering Physics, Tsinghua University, Beijing, China; Duke Cancer Institute, United States of America

## Abstract

**Background:**

Single nucleotide polymorphisms (SNPs) in DNA repair genes can alter gene expression and activity and affect response to cancer treatment and, correspondingly, survival. The present study was designed to evaluate the utility of the XRCC1 Arg399Gln and ERCC1 Cys8092Ala SNPs, measured in pretreatment biopsy samples, as predictors of response to radiotherapy in patients with non-metastatic nasopharyngeal carcinoma (NPC).

**Materials and methods:**

The study included 75 consecutive patients with stage II-IVA-B NPC. XRCC1 Arg399Glu and ERCC1 Cys8092Ala SNPs were identified from paraffin-embedded biopsy specimens via Sanger sequencing. Expression of p53 and pAkt protein was analyzed by immunohistochemical staining. Potential relationships between genetic polymorphisms and progression-free survival (PFS) were analyzed by using a Cox proportional hazards model, the Kaplan-Meier method, and the log-rank test.

**Results:**

Multivariate analysis showed that carriers of the ERCC1 8092 Ala/Ala genotype [hazard ratio (HR) 1.882; 95% confidence interval (CI) 1.031–3.438; *P* = 0.039] and heavy smokers (≥20 pack-years) carrying the XRCC1 Arg/Arg genotype (HR 2.019; 95% CI 1.010–4.036; *P* = 0.047) had significantly lower PFS rates. Moreover, combined positive expression of p53 and pAkt led to significantly increased PFS in subgroups carrying the XRCC1 Gln allele (HR 7.057; 95% CI 2.073–24.021; *P* = 0.002) or the ERCC1 Cys allele (HR 2.568; 95% CI 1.056–6.248; *P* = 0.038).

**Conclusions:**

The ERCC1 Cys8092Ala polymorphism is an independent predictor of response to radiotherapy for NPC, and the XRCC1 Arg399Glu mutation combined with smoking status seems to predict PFS as well. Our results further suggest a possible correlation between these genetic polymorphisms and p53 protein status on survival.

## Introduction

Radiation is an important component of treatmen for nasopharyngeal carcinoma (NPC). However, predictors of whether a particular tumor will respond to radiation therapy have not been identified. The biological parameters involved in carcinogenesis and determining response to radiation may have a strong impact on the choice of type and duration of radiotherapy. DNA repair pathways, for example, aid in maintaining genetic stability and preventing cancer development, but they also represent a potential mechanism of resistance to DNA-damaging radiotherapy. Polymorphisms in DNA repair genes provide the genetic basis for various DNA repair capabilities (DRC). For example, Arg399Gln (R399Q) in XRCC1 is a well-studied single nucleotide polymorphism (SNP) located in the BRCT1 domain, which is essential for PARP1-mediated recruitment of XRCC1 upon DNA damage. Substitution of arginine for glutamine is thought to cause loss of a secondary structure feature, such as an α-helix, critical for correct protein-protein interactions in the BRCT1 domain, thereby compromising DRC [1]. The 399Gln allele of XRCC1 may also directly alter mRNA expression [2]. The number of variant alleles in the XRCC1 R399Q genotypes has been correlated with prolonged cell cycle delays after ionizing radiation, resulting in hypersensitivity to ionizing radiation in women with breast cancer (*P* = 0.001) [3]. Hu *et al*. [4] suggested that the XRCC1 399Gln allele favorably prolongs delay of the cell cycle phase G_2_. Moreover, patients with the 399Gln allele exposed to ionizing radiation (either X-rays or γ-rays) had a higher frequency of micronuclei according to relative to those with the wild-type 399Arg allele [5]. Lunn et al. [6] detected higher levels of aflatoxin B1 adducts in patients with the XRCC1 Arg399Gln polymorphism and suggested that this may result in deficient DNA repair capacity. Theoretically, polymorphisms promote radiation sensitivity through inducing inefficient DRC and thereby enhancing cellular response to radiotherapy.

Excision repair cross complementing group 1 (ERCC1) is a highly conserved enzyme that is required for the incision step of nucleotide excision repair [7], [8]. The 8092 Cys/Ala polymorphism (C8092A) is located on the 3′ untranslated region of the ERCC1 gene [9], which has been implicated in the translational repression of ERCC1 mRNA [10], [12] although no functional differences have been described [11]. Potential relationships between SNPs of the ERCC1 gene such as Cys8092Ala and prognosis have been analyzed in patients with various types of cancer treated with cisplatin, with conflicting results [13]–[23]. However, studies of potential relationships between patients with ERCC1 Cys8092Ala and response to radiotherapy have been limited.

The ability to predict tumor radiosensitivity in patients with cancer before treatment is begun would allow radiotherapy to be tailored to each individual to optimize the effectiveness and minimize the toxicity of ionizing radiation in clinical practice. Most studies of SNPs in NPC have examined their relationship with susceptibility to cancer or to radiotherapy-induced toxicity. We speculate that the influence of smokinging on the prognosis of NPC is related to the polymorphyme of genes ERCC1,XRCC1 which are responsible for the DNA damage and repairing. However, the relationship between SNPs and response to radiotherapy has not been widely analyzed. We undertook the study described here in an attempt to fill that gap, and our findings clearly indicate that SNPs in ERCC1 and XRCC1 have an important role in the response of NPC to radiotherapy.

## Materials and Methods

### Patient selection

This retrospective analysis included 75 consecutive patients with locoregional NPC seen from January 2008 through October 2009 at the Department of Radiation Oncology of Hunan Provincial Tumor Hospital. Inclusion criteria were having biopsy-proven, previously untreated NPC of American Joint Committee on Cancer (AJCC)/International Union Against Cancer (UICC) stage II, III, or IVA-B. Other criteria included age >18 years, Han Chinese ethnicity, and an Eastern Cooperative Oncology Group performance status score of 0 or 1. Exclusion criteria included the presence of distant metastasis and other concomitant malignant disease. The study was approved by the Clinical Research Ethics Committee of the Hunan Province Cancer Center, and written informed consent was obtained from all patients.

### Pretreatment evaluation

All patients were evaluated with complete physical examinations, fiberoptic nasopharyngoscopy, magnetic resonance imaging (MRI) of the head and neck, chest X-ray, abdominal ultrasonography, and bone scans.

### Treatment

The primary tumor and neck lymph nodes were treated with megavoltage photons (6 MV). Radiation was administered five times per week at a dose of 2 Gy/day. The accumulated radiation dose was 68–72 Gy to the primary tumor, 60–62 Gy to involved areas of the neck, and 50 Gy to uninvolved areas. Concurrent chemoradiotherapy, consisting of 100 mg/m^2^ cisplatin on days 1, 22, and 43 during the radiotherapy, was administered to 39 patients (52%).

### Endpoints

The primary endpoint was progression-free survival (PFS), defined as the interval from the day of enrollment to the date of first documented relapse, which was categorized as locoregional (primary site or regional nodes) failure or distant metastases, or to the date of the last follow-up visit.

### Sample preparation, DNA extraction and quantification of DNA yield

Tissue specimens were collected from all patients? before treatment. From each sample, eight 10-µm sections were deparaffinized by using xylene and ethanol and digested with proteinase K for 24 h at 55°C. Subsequently, the enzyme was inactivated by boiling for 10 min before being mixed with absolute ethyl alcohol. Genomic DNA was purified by using ion-exchange columns and maintained at −20°C before use as described below.

### DNA amplification and genotyping procedures

The nested PCR method was used to amplify target fragments. The PCR reaction was performed in three steps as follows: 5 min at 95°C, followed by 35 cycles of 30 s at 95°C, 30 s at 55°C, and 40 s at 72°C, and 10 min at 72°C. Amplified products were identified via electrophoresis before Sanger sequencing using BigDye Terminator v3.1 (Life Technologies, Carlsbad, CA) on an Applied Biosystems 3130XL Genetic Analyzer. All PCR reactions and sequencing analyses were performed twice to verify the results.

### Immunohistochemical studies

Sections of NPC tissue obtained from all 75 patients were stained for immunohistochemical analysis by using standard techniques. Briefly, tissue sections were deparaffinized in xylene, rehydrated in a graded ethanol series, and treated with antigen retrieval solution (10 mmol/L sodium citrate buffer, pH 6.0). Next, sections were incubated with mouse anti-human p53 antibody (1∶200 dilution) overnight at 4°C and 1∶1,000 diluted biotinylated secondary antibody, followed by avidin-biotin peroxidase complex (all from DAKO, Carpinteria, CA), according to the manufacturer's instructions. Finally, tissue sections were treated with 3′,3′-diaminobenzidine (Sigma) until the development of a brown color and counterstained with Harris modified hematoxylin. Primary antibodies were omitted in the negative controls. The procedure for pAkt staining was similar to that for p53 staining, except that the anti-p53 antibody was replaced with anti-pAkt (Thr308) (Santa Cruz Biotechnology, Inc.). Sections were blindly evaluated by two investigators to provide a consensus on staining patterns. Both staining intensity and positive areas were recorded. A semiquantitative scoring criterion for immunohistochemistry was used in which both staining intensity and positive areas were recorded according to the method of Hara et al. [24]. At least 10 high-power fields were selected randomly, and >1,000 cells were counted for each section. Staining intensity was graded as follows: 0: no staining, 1+: mild staining, 2+: moderate staining, and 3+: intense staining. Staining area was scored as follows: 0: no staining in any microscopic field, 1+: <30% of tissue stained positive, 2+: between 30% and 60% stained positive, and 3+: >60% stained positive. The minimum score when summed (extension + intensity), was 0, and maximum score was 6. For pAkt, a combined staining score (extension + intensity) of ≤3 was considered negative (low staining), and that between 4 and 6 was considered positive. For p53, a combined staining score (extension + intensity) of ≤4 was considered negative (low staining), and that between 5 and 6 was considered positive.

### Posttreatment Follow-up

The follow-up period ended on October 31, 2012, for a median follow-up period of 25 months (range 5–46 months) for all patients. After treatment, patients were followed up at least every 3 months during the first year and then every 6 months thereafter until disease progression (recurrence or distant metastasis). Local recurrence was diagnosed with fiberoptic endoscopy and biopsy or MRI when the nasopharynx and skull base showed progressive bone erosion or soft tissue swelling. Regional recurrences were diagnosed based on clinical examination of the neck and, in doubtful cases, with fine needle aspiration or MRI of the neck. Distant metastases were diagnosed based on clinical symptoms, physical examination, and imaging methods, including chest radiography, abdominal ultrasonography, whole body bone scans, CT scans, and MRI.

### Statistical analysis

The Kaplan-Meier method was used to estimate survival curves, and the log-rank test used to compare survival times between subgroups. Multivariate analyses with Cox regression were used to assess the significance of clinical variables with adjustment for age, sex, clinical disease stage, receipt of concurrent chemotherapy, and smoking status. All analyses were done with SPSS 17.0 (SPSS). All statistical tests were two-sided, and a value of *P*<0.05 considered statistically significant.

## Results

Patient characteristics are summarized in Table 1. The study included 57 men and 18 women, for a male-to-female ratio of 3.2∶1, and the median age was 45 years (range, 22–72 years). All patients had World Health Organization (WHO) grade 2–3 NPC, 16 with stage II disease, 44 stage III, and 15 stage IVA–B disease. During the follow-up period, 22 (29%) patients had locoregional relapse and 21 (28%) had distant metastasis. No patients died during follow-up. The 3-year PFS rate was 42.7%.

**Table 1:** Patient demographics and treatment characteristics.

Characteristics	Number of patients (%)
Age, y	22–72
Median age	45
Gender	
Male	57(76.0)
Female	18(24.0)
Overall stage (AJCC)^a^	
II	16(21.3)
III	44(58.7)
IVa-b	15(20.0)
T classification^a^	
T1-2	42(56.0)
T3-4	33(44.0)
N classification^a^	
N0	16(21.3)
N1-3	59(78.7)
Concurrent chemotherapy	
No	36(48.0)
Yes	39(52.0)
Adjuvant chemotherapy	
No	35(46.7)
Yes	40(53.3)
Smoking status (Pack-years)	
0	38(50.7)
>0,<20	16(21.3)
≥20	21(28.0)
≥30	16(21.3)

a 7^th^ American Joint Committee on Cancer/International Union Against Cancer staging system.

**Figure 1:**
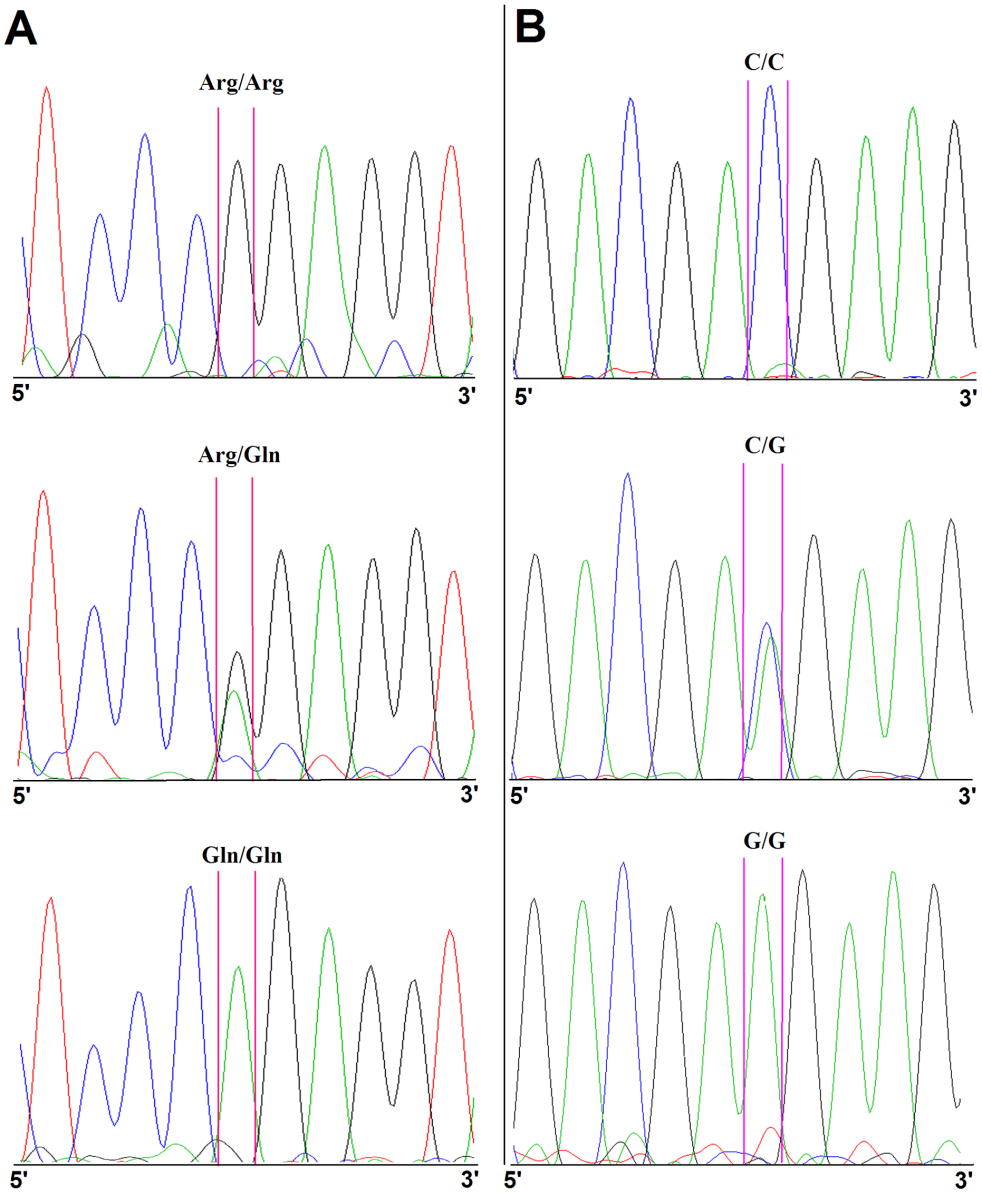
Typical raw data obtained using Sanger sequencing instruments. The area between the two vertical lines indicates the resulting genotypes of XRCC1 Arg 399Gln (A) and ERCC1 Cys8092Ala (B).

XRCC1 Arg399Gln and ERCC1 C8092A polymorphisms were clearly distinguished via Sanger sequencing. Typical sequencing peaks are depicted in Figure 1. The frequency of the Cys allele was 56.7% for ERCC1 8092 Cys>Ala polymorphism and that of the Arg allele was 76% for the XRCC1 Arg399Gln polymorphism. No significant associations were found between XRCC1/ERCC1 polymorphisms and clinical variables (data not shown). Expression of p53 and pAkt similarly were not associated with either the XRCC1 Arg399Gln or the ERCC1 Cys8092Ala polymorphisms (data not shown).

**Figure 2:**
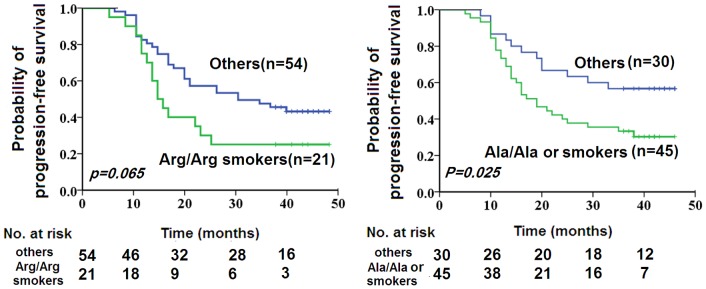
Kaplan-Meier progression-free survival (PFS) curves according to SNPs of DNA repair genes. A represents PFS curves according to the combination of XRCC1 Arg399Gln and smoking status; B shows PFS curves according to ERCC1 Cys8092Ala and smoking status.

Kaplan-Meier PFS curves generated according to the XRCC1 Arg399Gln genotype combined with smoking status are depicted in Figure 2A. Log-rank analysis revealed slightly shorter time to disease progression in smokers carrying the XRCC1 399 Arg/Arg genotype relative to other groups, although not to a significant extent (20.1 months vs. 30.2 months, *P* = 0.065). In multivariate analysis with a Cox proportional hazards model, hazard ratios (HRs) for disease progression (distant metastases and local recurrence) were significantly higher for smokers carrying the XRCC1 399 Arg/Arg genotype after adjustment (HR 2.019, 95% confidence interval [CI] 1.010–4.036; *P* = 0.047).

Log-rank analysis revealed significantly shorter time to disease progression in subjects carrying the ERCC1 Ala/Ala genotype versus those carrying the 8092 Cys allele (22.8 months vs. 30.9 months, *P* = 0.033). In multivariate analysis with a Cox proportional hazards model, HR for disease progression (distant metastases and local recurrence) was significantly higher for Ala/Ala genotype carriers after adjustment (HR 1.882, 95% CI 1.031–3.438; *P* = 0.039). Kaplan-Meier PFS curves generated according to the ERCC1 Cys8092Ala genotypes and smoking status are shown in Figure 2B. Cys carriers who never smoke showed better results than others(33.6 months vs. 25.3 months,*P* = 0.025).

Next, the effects of smoking status on risk of disease progression (local recurrence and distant metastases) associated with the XRCC1 Arg399Gln and ERCC1 Cys8092Ala genotypes were subjected to stratification analysis. Results are shown in Table 2. In multivariate analysis with a Cox proportional hazards model, the HR for disease progression was significantly higher for heavy smokers in the subgroup with the XRCC1 399 Arg/Arg (HR 2.833, 95% CI 1.080–7.429; *P* = 0.034) and ERCC1 8092 Cys/Cys or Cys/Ala genotypes (HR 3.515, 95% CI 1.373–8.995; *P* = 0.009). No significant association between smoking status and PFS was detected in subgroups with the XRCC1 399 Gln/Gln or Arg/Gln or the ERCC18092 Ala/Ala genotypes.

**Table 2:** Log-rank and proportional hazard analysis (Cox method) for progression-free survival (PFS) related to smoking status affected by polymorphism of XRCC1 Arg399Gln and ERCC1 8092 Cys>Ala.

smoking status (Pack-years)	Genotypes	Log-rank analysis		Cox-regression		
		Mean survival time(months)	*p*	HR	95% CI	*p*
	*XRCC1 Arg399Gln*					
≥20	Arg/Gln+Gln/Gln	22.75±5.25	0.278	1.350	0.351–5.190	0.622
<20	Arg/Gln+Gln/Gln	29.75±3.45	—	1	reference	—
≥20	Arg/Arg	20.15±3.43	**0.024**	**2.833**	**1.080–7.429**	**0.034**
<20	Arg/Arg	32.12±2.59	—	1	reference	—
	*ERCC1 8092 Cys>Ala*					
≥20	Cys/Cys +Cys/Ala	19.92±3.58	**0.004**	**3.515**	**1.373–8.998**	**0.009**
<20	Cys/Cys +Cys/Ala	34.28±2.37	—	1	reference	—
≥20	Ala/Ala	23.25±5.01	0.863	0.680	0.167–2.777	0.591
<20	Ala/Ala	22.53±3.17	—	1	reference	—

**Figure 3:**
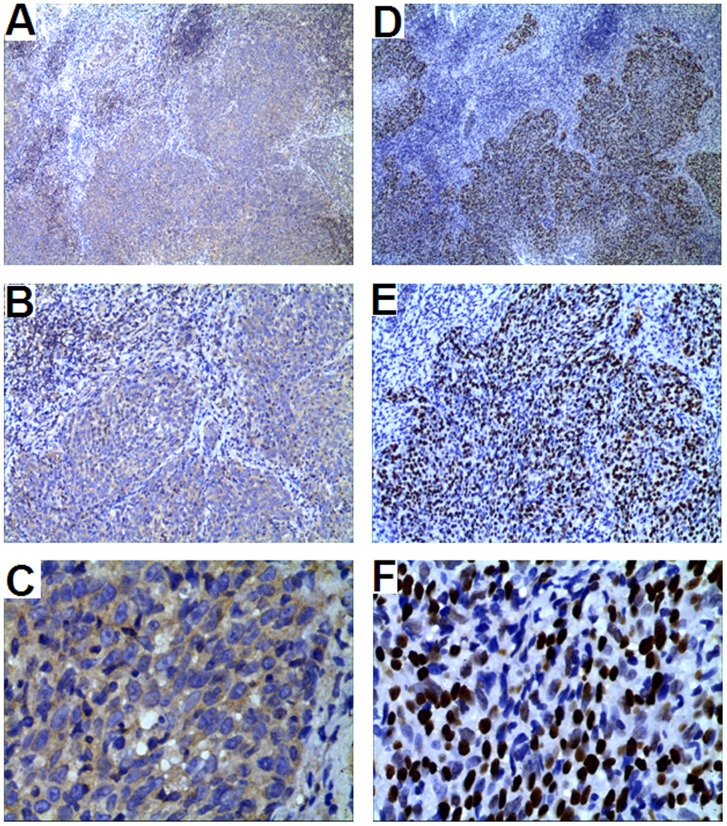
Immunohistochemical analysis of p53 and pAkt expression in NPC tissues. Brown cytoplasmic staining of tumor cells, indicative of positive pAkt, shown in A (x40), B (x100), and C (x400); Brown nuclear staining of tumor cells, indicative of positive p53, shown in D (x40), E (x100), and F (x400).

Representative results of p53 and pAkt immunohistochemical analysis are shown in Figure 3. The effects of combined p53 and pAkt protein expression on risk of disease progression (local recurrence and distant metastases) were examined via stratification analysis according to the XRCC1 Arg399Gln and ERCC1 Cys8092Ala genotypes (Table 3). In multivariate analysis with a Cox proportional hazards model, the HR for disease progression was significantly higher for patients with combined positive expression of both p53 and pAkt in subgroups carrying XRCC1 399 Gln/Gln or Arg/Gln genotypes (HR 7.057, 95% CI 2.073–24.021, *P* = 0.002) and ERCC1 8092 Cys/Cys or Cys/Ala genotypes (HR 2.568, 95% CI 1.056–6.248, *P* = 0.038). No significant association between protein expression and PFS was detected in subgroups carrying the XRCC1 399 Arg allele or the ERCC1 Ala/Ala genotype.

**Table 3:** Log-rank and proportional hazard analysis (Cox method) for progression-free survival (PFS) related to p53 and pAkt protein expression affected by polymorphism of XRCC1 Arg399Gln and ERCC1 8092Cys>Ala.

P53 and pAkt protein expression	Genotypes	Log-rank analysis		Cox-regression		
		Mean survival time(months)	*p*	HR	95% CI	*p*
	*XRCC1 Arg399Gln*					
Both positive	Arg/Gln+Gln/Gln	15.70±3.44	**0.001**	**7.057**	**2.073–24.021**	**0.002**
others*	Arg/Gln+Gln/Gln	34.61±3.27	—	1	reference	—
Both positive	Arg/Arg	26.37±2.65	0.686	0.759	0.257–2.237	0.617
others*	Arg/Arg	29.64±4.14	—	1	reference	—
	*ERCC1 8092 Cys>Ala*					
Both positive	Cys/Cys +Cys/Ala	24.50±3.89	0.066	**2.568**	**1.056–6.248**	**0.038**
others*	Cys/Cys +Cys/Ala	32.58±2.65	—	1	reference	—
Both positive	Ala/Ala	21.50±4.72	0.898	1.022	0.326–3.202	0.970
others*	Ala/Ala	23.47±3.28	—	1	reference	—

*Note*: *Means either P53 negative or pAkt negative.

## Discussion

Our results showed that the presence of the XRCC1 399Arg allele, in combination with smoking, was associated with poor PFS in patients with NPC treated with curative radiotherapy (Figure. 2A). Earlier research suggests that the 399Gln allele of XRCC1 directly affects DRC [1]–[5], but other evidence shows that the Arg399Gln polymorphism of XRCC1 has no adverse effects on the DNA repair pathway [25], [26], [27]. The results collectively indicate that DNA repair capacity arising from low-penetrance genes, including the XRCC1 polymorphism, may be modified by other genetically determined or environmental risk factors that correlate with response of NPC to radiotherapy. Although XRCC1 Arg399Gln did not affect response to radiotherapy as a dependent predictor in the current study, we speculate that this polymorphism has a key role in smoking-induced radioresistance.

The precise mechanisms by which cigarette smoking and DNA repair polymorphisms affect response to radiotherapy remain unknown. Cigarette smoking is reported to induce DNA damage as well as stimulate DNA repair [28]. One possible explanation is that the high-DRC phenotype stimulated by smoking may be influenced by the genotype of XRCC1 399Gln carriers. Several reports have shown that smokers tend to have more proficient DRC than non-smokers [29], [30]. Moreover, expression levels of several DNA repair genes in peripheral lymphocytes have been shown to be higher in smokers than in non-smokers [31]. Cigarette smoke contains many genotoxic agents and carcinogens, such as nitrosamine 4-(methylnitrosamino)-1-(3-pyri-dyl)-1- butanone (NNK) and reactive oxygen species. XRCC1 399Gln/Gln genotype carriers reportedly have decreased capacity to repair NNK-induced sister chromatid exchange [32], [33]. Furthermore, increased 8-hydroxydeoxyguanine levels seem to be suppressed, and consequent oxidative damage in DNA repaired, through the base excision repair pathway [34]. Because the XRCC1 protein is known to participate in this pathway with DNA ligase III, PARP, and POL (DNA polymerase) [35], it is biologically plausible that amino acid substitution at codon 399 also influences the repair of oxidatively damaged DNA. Because the common allele seems to be associated with higher DRC stimulated by genotoxic agents in cigarette smoke, it is reasonable to assume that smokers carrying the XRCC1 399 Arg allele show increased repair of DNA damage induced by ionizing radiation relative to other groups. A second possible explanation is that high exposure to tobacco carcinogens generates potentially radioresistant preneoplastic cells. Lower DRC may be associated with a higher rate of apoptosis of preneoplastic cells, whereas effective DNA repair decreases the rate of apoptosis. Consequently, NPC in smokers who carry the XRCC1 Arg/Arg genotype and have higher DRC may be more likely to display the radioresistant characteristics of preneoplastic cells. However, prospective clinical studies are needed to validate these suppositions.

Notably, we observed reduced response to radiotherapy in heavy smokers (≥20 pack-years) for Arg allele carriers but not for Gln/Gln genotype carriers (Table 2), which supports the assumption that the Arg allele is essential for smoking-induced radioresistance. The exact mechanism underlying gene–smoking associations for the XRCC1 399 Arg/Gln polymorphism warrants further investigation.

In the present study, NPC patients carrying the ERCC1 8092Ala/Ala genotype had poorer outcomes than those with ERCC1 8092A/C or ERCC1 8092 Cys/Cys genotypes. Our findings are consistent with previous findings for other types of cancer. In studies of non small-cell lung cancer, the the variant allele (Cys→Ala) tends to predict worse outcomes [13], [14], [15]. In other tumors treated with platinum-based chemotherapy, such as ovarian cancer, malignant mesothelioma, or advanced squamous cell carcinoma of the head and neck, association of the ERCC1 8092Ala allele with poor survival has been consistently reported [16], [17], [18]. Other investigators [19], [20] have demonstrated that the presence of at least one 8092Ala allele is associated with improved outcomes in patients with advanced non-small cell lung cancer. Similarly, association of the 8092Ala allele with improved overall survival has been reported for patients with esophageal cancer treated with cisplatin [21]. However, several other studies suggest that the C8092A polymorphism does not correlate with PFS after platinum-based chemotherapy [22], [23]. The discrepancies in these results could be attributed to many factors, most likely differences in the types of malignancies, ethnicity, and SNP distribution frequencies.

The ERCC1 8092 Cys>Ala polymorphism may be a contributory factor in the development of NPC in the Chinese population; one study indicated that individuals with the 8092Cys allele had a 1.4-fold increase in the risk of developing NPC [36]. The ERCC1 Cys8092Ala polymorphism has been suggested to independently predict PFS in patients with NPC treated with cisplatin-based chemotherapy; compared with patients carrying the Cys/Cys genotype, those with Cys/Ala or Ala/Ala genotype were shown to have increased risk of disease progression while on such chemotherapy [37]. Because low DNA repair capacity leads to higher genomic instability, and consequently higher cancer susceptibility and improved response to DNA-damaging treatment, the ERCC1 8092Ala allele probably has higher DRC than the 8092Cys allele in NPC. To complement studies examining the association of polymorphisms with cancer susceptibility and radiation-induced toxicity, we investigated their relationship with response to radiotherapy. In multivariate analysis adjusted for the most important clinical and biologic characteristics, the ERCC1 8092A/A genotype was independently associated with shorter time to progression (Figure. 2B). In addition to its involvement in the nucleotide excision repair pathway, ERCC1 is involved in other repair mechanisms, such as interstrand crosslink repair and homologous recombination, the latter being one of the major repair pathways for double-strand breaks [38]. Double-strand breakage has been established as the critical insult by which ionizing radiation kills cells [39]. These observations may aid in explaining why ERCC1 8092 Cys>Ala is an independent predictor of prognosis in patients with NPC treated with curative radiotherapy.

We further investigated whether smoking history modifies the effect of the ERCC1 Cys8092Ala polymorphism on survival. In stratification analysis by ERCC1 8092 polymorphisms, we observed an adverse effect of heavy smoking on survival in patients with NPC carrying the ERCC1 8092C allele but not in those with the ERCC1 8092Ala/Ala genotypes (Table 2). The ERCC1 8092Ala allele may be correlated with other clinical features that lead to treatment resistance, thereby obscuring any subtle effects of heavy smoking in altering DRC.

The molecular mechanisms underlying the effects of ERCC1 8092Cys>Ala polymorphism on survival in patients with NPC remain largely unexplained. At present, it is unclear whether the polymorphism increases the DRC or vice versa. Therefore, in addition to alternative splicing of ERCC1, research focused on determining the relationship between ERCC1 polymorphisms and mRNAs is essential.

We further determined the effects of SNPs in DNA repair genes on p53 protein status as predictors of radiosensitivity in NPC. Inactivation of the p53 protein in NPC can be easily detected via immunohistochemical staining because of its extended half-life [40]; indeed, p53 protein expression has been established as a marker of radioresistance both *in vivo* and *in vitro* [41]. However, multiple conflicting clinical reports exist, of which only a few have reported p53 overexpression as being associated with poor survival [42], [43], [44]. Akt is a downstream effector of PI3 kinase in NPC, possibly associated with aggressive tumor behavior and poor survival in patients with NPC [45]; pAkt inhibits p53 function in a Mdm2-dependent manner [46]. To ascertain the precise function of p53 in this context, we evaluated both p53 and pAkt expression with immunohistochemical staining. In our experiments, neither p53 alone nor combinations of p53 and pAkt were correlated with survival in NPC (data not shown). Nevertheless, combined positive expression of p53 and pAkt was associated with poorer outcomes only in patients carrying a favorable genotype (XRCC1 399Gln allele or ERCC1 8092Cys allele carriers). This association was insignificant in patients carrying an unfavorable genotype (XRCC1 399Arg/Arg or ERCC1 8092Ala/Ala). One possible explanation for this is that the pro-apoptotic function of p53 is minimized or offset by the effects of increasing DRC caused by the unfavorable genotypes of XRCC1 or ERCC1.

In conclusion, we have demonstrated that XRCC1 399Arg, in combination with heavy smoking, is significantly associated with poorer prognosis and that ERCC1 8092C alone is associated with better prognosis in patients with NPC treated with radiotherapy. These findings are in agreement with the possibility that effective DNA repair reduces the likelihood of tumor response to radiotherapy. We further examined the effects of SNPs in DNA repair genes on p53 protein status as predictors of radiosensitivity in NPC. However, further investigation of the underlying molecular mechanisms to explain how these polymorphisms affect response to radiotherapy and prospective clinical studies in patients with NPC are needed to validate our results.
